# The Boss’s Long Arm: The Differential Impact of Authoritarian and Benevolent Leadership on Spousal Family Satisfaction

**DOI:** 10.3389/fpsyg.2021.780030

**Published:** 2021-11-25

**Authors:** Lei Yao, Minya Xu, Ekin K. Pellegrini

**Affiliations:** ^1^Business School, Inner Mongolia University of Finance and Economics, Hohhot, China; ^2^Guanghua School of Management, Peking University, Beijing, China; ^3^College of Business Administration, University of Missouri-St. Louis, St. Louis, MO, United States

**Keywords:** authoritarian leadership, benevolent leadership, work–family conflict, work-family facilitation, spousal need for control, spousal family satisfaction

## Abstract

The extant studies on leadership are replete with employee, coworker, and leader outcomes, however, research is still nascent on leadership’s crossover into employees’ family members’ lives. To examine leadership’s impact on the work–family interface, we draw on conservation of resources theory (COR) and crossover theory and investigate how authoritarian leadership and benevolent leadership affect spousal family satisfaction. We examine the mediating influence of work–family conflict (WFC) and work-family facilitation (WFF), and the moderating impact of spouses’ need for control. Our model was tested with multisource, mutiwave data from 207 Chinese married dyads. The results suggest that, as expected, the positive relationship between benevolent leadership and spousal family satisfaction is fully mediated by WFF, and the negative relationship between authoritarian leadership and spousal family satisfaction is fully mediated by WFC. Findings further suggest that the negative relationship between employee WFC and spousal family satisfaction is stronger for spouses with a higher need for control. Thus, authoritarian leadership, through its negative influence on WFC appears to be universally detrimental for spousal family satisfaction, however, even more so for spouses with a higher need for control. These results underscore the importance of acknowledging leadership’s impact at work reaching far beyond the job incumbent.

## Introduction

In this game of life, your family is the court and the ball is your heart. No matter how good you are, no matter how down you get, always leave your heart on the court.

–Kwame Alexander, *The Crossover*

Over the past 2 decades, there is increasing research interest on the well-being of employees and their families (Westman, 2001; Hobfoll et al., 2018; Thompson et al., 2021). This is not surprising, as sustainable success of an organization is inevitably intertwined with its ability to achieve high-quality performance and a healthier workforce. Previous longitudinal research has even suggested employee psychological well-being as an antecedent of employee job performance (Wright and Cropanzano, 2004).

Previous research suggests employees’ experiences at work may affect their partners at home (Bakker et al., 2009b). One important factor influencing employees’ attitudes and behaviors is leadership behavior, the effects of which may subsequently cross over to employees’ partners at home. Liao et al. (2015) found that ethical leadership may positively influence employees to engage in similarly ethical behaviors in the family context, which may in turn boost spousal family satisfaction. Yang et al. (2018) found servant leadership and job social support to be positively related with employees’ organization-based self-esteem, which in turn positively influenced family satisfaction and quality of family life experienced by spouses. Further, Zhou et al. (2019) found a positive relationship between authentic leadership and employee’s work-to-family positive spillover, which in turn positively influenced romantic love as rated by employees’ spouses. Despite these consistent findings, research on the influence of leadership behaviors on employees’ spouses remains in its infancy. Further, limited existing research has exclusively focused on the bright side of leadership (i.e., supportive leader behaviors), and completely neglected leadership behaviors that may have a negative spillover effect on spouses.

Authoritarian leadership and benevolent leadership emerge as the most prevalent leadership styles in collectivistic cultures with high power distance, such as Asia, the Middle East, and South America (Farh and Cheng, 2000; Martinez, 2003; Pellegrini et al., 2010; Schaubroeck et al., 2017). Despite mounting evidence from these business contexts supporting the widespread presence of these two leadership styles and their significant influence on a wide spectrum of employee attitudes and behaviors, such as organizational commitment, organizational citizenship behavior, creativity, leader-member exchange (LMX), and job performance (e.g., Pellegrini and Scandura, 2006; Wang and Cheng, 2010; Chan et al., 2013; Chan, 2014; Schaubroeck et al., 2017), existing work has largely failed to consider their influence outside the work context, specifically on employees’ family members. This is a surprising omission given that, specifically for dual-career couples, the spillover from employees’ work context to family may have extended repercussions to spouses’ work attitudes in their own business settings.

In this study, based on conservation of resources theory (COR; Hobfoll, 1989) and crossover theory (Westman, 2001), we explore the influence of authoritarian leadership and benevolent leadership on spousal family satisfaction through the mediating influence of work–family conflict (WFC) and work-family facilitation (WFF). Work–family conflict is a form of inter-role conflict in which the demands of work and family roles are incompatible and participation in one role becomes more difficult because of participation in the other role (Greenhaus and Beutell, 1985). On the other hand, work-family facilitation is defined as a form of role accumulation in which the resources of work and family roles are complementary and participation in work role makes it easier to perform the other role (Grzywacz and Marks, 2000; Grzywacz and Bass, 2003; Wayne et al., 2007). Voydanoff (2004) suggests that in work–family conflict, demands hinder the performance of work and family roles, whereas in work-family facilitation, resources enhance their performance.

Authoritarian leaders, due to their emphasis on authority and strong discipline, can create excessive work obligations, leaving employees unable to fully satisfy their family roles. In this study, we examine authoritarian leadership’s negative influence on spousal family satisfaction, through its impact on work–family conflict. At the same time, we acknowledge that when employees experience work–family conflict, spouses who desire a more predictable and controlled family environment may be more negatively affected. Therefore, we also examine the moderating role of spouses’ need for control in the negative relationship between work–family conflict and spousal family satisfaction.

Benevolent leaders, on the other hand, not only provide on-the-job support at work, but they also provide support for employees’ family lives (Cheng et al., 2000; Pellegrini and Scandura, 2008), which may help employees gain resources to better fulfill their family obligations. Accordingly, we examine the positive influence of benevolent leadership on spousal family satisfaction, through its positive impact on work-family facilitation.

The current study advances our knowledge of the spillover effect of leadership in three ways. First, this is the first study to investigate the differing effects of authoritarian and benevolent leadership on employees’ family context. This is particularly important in collectivist cultures high in power distance since employees tend to comply with their leaders’ expectations of their time and resources, both on and off the job. Therefore, in such work contexts, leadership effects may play even a more vital role in affecting employees’ off-the-job lives.

Second, with a theoretical foundation in COR and the crossover theory, our research demonstrates the influential and central roles of work–family conflict and work-family facilitation in the under-studied link between leadership and spousal family satisfaction. Overall, our results indicate that both the demands from authoritarian leadership and the resources from benevolent leadership may extend to employees’ spouses, which enrich our knowledge of the influencing mechanisms at work for the work-family interface, and contribute to a better understanding of the triggers of work–family conflict and work-family facilitation. Third, we establish that work–family conflict has differing effects on spousal family satisfaction at different levels of spouse’s need for control. Unlike previous crossover research which largely focused on employee resources, such as family identity salience (Lu et al., 2016), empathy (Liu and Cheung, 2015), and identification with leader (Liao et al., 2015) playing a buffering role for challenging situations at work, we find that a spouses’ high need for control amplifies the negative impact of employee work–family conflict, and leads to significantly lower spousal family satisfaction when employees experience work–family conflict due to authoritarian leadership. Findings extend the current work–family literature by showing the importance of integrating spouses’ characteristics in modeling the crossover effects of employee work experiences and their impact on spouses.

**Figure 1:**
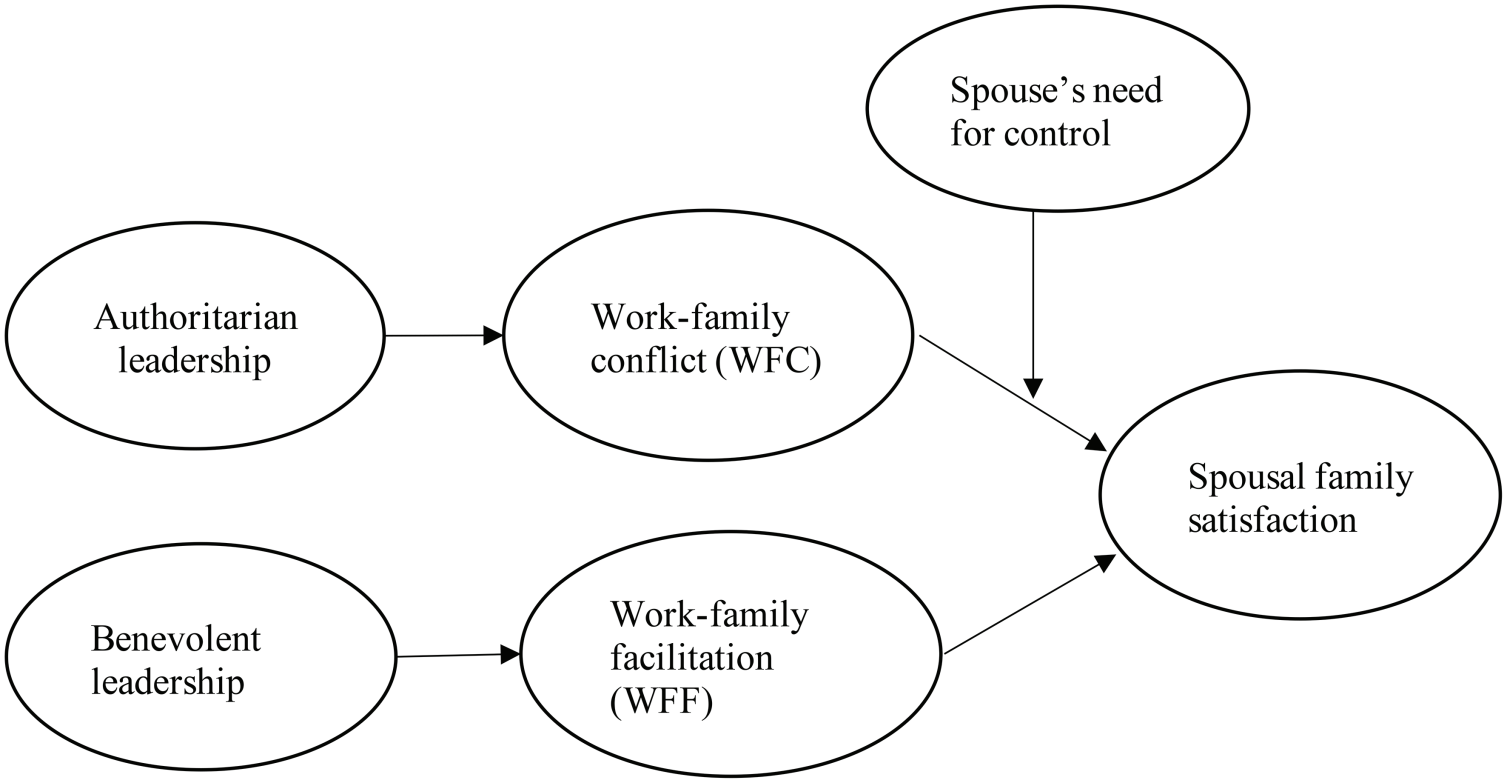
Research framework.

To examine our research model, we collected data from Chinese employee-spouse dyads using a multi-wave research design with three time periods. We chose to collect data from Chinese companies, because authoritarian leadership and benevolent leadership are highly prevalent in China, and a greater understanding of employee experiences associated with these two types of leadership could help business organizations build practices that better promote the well-being of employees and their spouses (Figure 1).

## Theoretical Background

COR theory suggests that individuals have a tendency to preserve, protect, and acquire resources. Resources refer to anything that helps individuals achieve their goals, such as time, energy, efficiency, knowledge, and material (Hobfoll, 1989; Hobfoll et al., 2018). Employees feel stressed when they face resource loss threats and actual resource loss, and they strive to acquire resources to protect against future resource loss (Halbesleben et al., 2014). We use COR theory to inform how authoritarian leadership and benevolent leadership affect employees’ family domain. Given the authority and strict control authoritarian leaders exert over employees, it is reasonable to argue that authoritarian leaders may make employees consume more resources (e.g., time), and experience strain. As a result, employees may be unlikely to manage their family roles, and experience heightened work–family conflict (Greenhaus and Beutell, 1985; Bakker et al., 2008), which may subsequently affect their spouses’ family satisfaction. Additionally, spouses’ need for control necessitates a need to extend additional resources to exert greater control over family life, which may be a boundary condition in the relationship between employees’ work–family conflict and spouses’ well-being.

Given their holistic care for employees’ work and family lives, benevolent leaders are likely to equip employees with capital and developmental resources (e.g., positive emotions, job skills, etc.) that may help them better manage their family obligations (Greenhaus and Powell, 2006), which may ultimately positively affect spousal family satisfaction.

COR theory posits that employee experiences of strain and resources may transfer to family members (Westman, 2001). Previous research suggests that strain and resources may impact spouses through empathy and interpersonal interactions, such as social undermining or social support (Bakker and Demerouti, 2012). Spouses may internalize one another’s emotional state as their own (Westman, 2001). Accordingly, anxiety, exhaustion, life satisfaction, and relational satisfaction could be transferred between spouses (Westman, 2001; Demerouti et al., 2005; Bakker et al., 2009a). We use crossover theory to inform our investigation of the crossover effect of leadership impact from the employee (work–family conflict and work-family facilitation) to the spouse (spousal family satisfaction).

## Authoritarian Leadership and Work–Family Conflict

Authoritarian leaders exert power, control, and authority over their employees. They require employees’ absolute obedience and reprimand employees when they do not follow their commands (Cheng et al., 2000; Chan et al., 2013; Chen et al., 2014). Authoritarian leadership is manifested in leader’s unwillingness to empower employees, communicate with employees, and disregard suggestions from employees (e.g., Chan et al., 2013). Authoritarian leadership is regarded as a destructive leadership style that negatively affects employee performance and psychological resources (e.g., Chen et al., 2014).

Research identifies abusive supervision to be a common destructive leadership style in Western organizations, however, authoritarian leadership is distinct from abusive supervision (Tepper, 2000). First, abusive supervision devalues employees’ abilities and ignore their contributions, whereas authoritarian leadership emphasizes withholding information (the leader alone has access to relevant information), strictly controlling employees’ behavior, and instructing employees to perform according to the leader’s directions (Cheng et al., 2004). Second, they differ in their behavioral motivations. Authoritarian leaders seek to meet their own psychological needs for controlling and demonstrating their power, and they have no intention of deliberately harming their employees (Pellegrini and Scandura, 2008). In contrast, abusive leaders demonstrate hostile behaviors in a quest for satisfying their own private interests or passing their negative experiences to employees (Tepper, 2000). Thus, not all authoritarian leaders will exhibit abusive behavior (Chou et al., 2014).

As a display of their authority, authoritarian leaders often hide key work information from employees, and they reprimand employees when they feel short of meeting their job requirements (Farh and Cheng, 2000). Employees thus have to work with vague information, make errors, and waste considerable time. The behavioral controls imposed on employees by authoritarian leaders make it hard for employees to gain the resources that may help them decrease the negative effects of their high pressure work environment (Jackson and Schuler, 1985). Therefore, we expect that the authoritarian leadership will lead to a heightened employee work–family conflict.

*Hypothesis 1*: Authoritarian leadership is positively related to employee work-family conflict.

Based on COR theory, when employees experience work–family conflict under authoritarian leadership, they have to invest less time on the family domain. As a result, their spouses have to devote more time to meet family obligations, which may increase spousal stress, especially if the spouse also works outside of the home. A number of studies have emphasized that work obligations interfering with family roles negatively relate to family satisfaction (e.g., Frone et al., 1994; Boyar and Mosley, 2007). Further, employees feeling strained based on work–family conflict could transfer those feelings to their spouses. Employees may crossover their strain to their spouses through emotional diffusion (Bakker and Demerouti, 2009). Westman and Etzion (1995) found that military officers with high levels of burnout were more likely to trigger burnout in their spouses. Westman et al. (2004) state that anxiety from one spouse may directly crossover to the other spouse. Howe et al. (2004) found that depression could also crossover to spouses. These shared negative experiences may transfer employee’s negative attitudes to the whole family (Chesley, 2005).

When employees experience work–family conflict, they may initiate or exacerbate negative interactions with their spouses (Bakker and Demerouti, 2012). Wu et al. (2012b) suggest that when employees experience work–family conflict, they tend to display higher levels of family undermining behaviors (i.e., aggressive actions) toward their spouses. Shimazu, Bakker and Demerouti (2009) suggest that work–family conflict may increase social undermining and decrease social support. Employees experiencing work–family conflict are more likely to display increased marital hostility and decreased marital intimacy (Matthews et al., 1996; Bakker et al., 2008; Shimazu et al., 2009; Liu et al., 2013), which may negatively influence spousal family satisfaction. We thus propose the following hypothesis:

*Hypothesis 2*: Employee work-family conflict is negatively related to spousal family satisfaction.

Employees working for authoritarian leaders are highly unlikely to obtain adequate resources to recover from their strained work environments. Therefore, authoritarian leadership will likely trigger a heightened state of employee work–family conflict (Voydanoff, 2004), which may in turn negatively influence spousal family satisfaction. Thus, we propose the following full mediation hypothesis:

*Hypothesis 3*: Employee work-family conflict mediates the negative relationship between authoritarian leadership and spousal family satisfaction.

## Benevolent Leadership and Work-Family Facilitation

Benevolent leaders show holistic and individualized care for employees’ well-being in both work and non-work domains (Farh and Cheng, 2000; Wang and Cheng, 2010). Benevolent leadership includes personalized care and protection (Cheng et al., 2004). Benevolent leaders not only devote time and energy to mentor employees in workplace, but they also provide support to employee’s family similar to an elder family member, such as lending money or attending family weddings and funerals (e.g., Cheng et al., 2000; Aycan, 2006; Pellegrini and Scandura, 2008; Chan and Mak, 2012; Aycan et al., 2013).

Previous research has found positive effects of servant leadership on the quality of family life experienced by the employees’ spouses (Yang et al., 2018). Benevolent leadership is distinct from servant leadership in at least two aspects. First, benevolent leaders do not only pay attention to employee’s work performance and their care extends to employee’s family life as well, whereas servant leadership as conceptualized in the Western business context may regard leader’s attention to employee’s personal life as a violation of their privacy. Second, servant leaders put employee’s job needs first, even if that requires self-sacrifice on the leader’s part (Liden et al., 2008). Benevolent leaders, similar to servant leaders, also provide employees with instrumental and emotional support, but the relationship between leaders and employees is based on mutual benefits, and benevolent leaders expect employees to reciprocate leader’s care and protection with loyalty and respect (Farh and Cheng, 2000). Carlson et al. (2006) identify three types of work resources that promote work-family enrichment: development resources, affect resources, and capital resources. Development resources include skills, knowledge, and values. Affect resources refer to moods and attitudes. Capital resources are composed of economic, social, or health assets. Benevolent leaders provide employees with all these resources, as well as opportunities to learn from their mistakes and correct their wrong doings. When employees face work and life challenges, they can seek help from benevolent leaders (Farh and Cheng, 2000; Karakitapoğlu-Aygün et al., 2020). The extensive resources employees receive from benevolent leaders increase employee psychological resources, such as self-esteem and hope, which trigger positive emotions, satisfaction, affective trust, and high quality LMX relationships (Greenhaus and Powell, 2006; Wasti et al., 2011; Chen et al., 2014; Zhang et al., 2015). The positive emotions employees bring from work may transfer to their spouses (Carlson et al., 2006). Moreover, benevolent leaders promote employee performance (Chan and Mak, 2012; Wu et al., 2012a), which may bring more social and economic resources, benefiting their quality of family life. Greenhaus and Powell (2006) state that receiving instrumental resources (e.g., skills, psychological resources, and material resources) at work may facilitate employees’ family lives. We thus propose the following hypothesis:

*Hypothesis 4*: Benevolent leadership is positively related to employee work-family facilitation.

When employees experience work-family facilitation, they have the resources to be both positive and efficient in the home domain, which may provide considerable support to spouses and have a positive impact on spousal family satisfaction (Wayne et al., 2007; Bakker and Demerouti, 2012). According to crossover theory, employees’ positive work experiences may directly transfer to their spouses *via* interpersonal interactions in daily life (Demerouti et al., 2005; Westman et al., 2009). When employees experience work-family facilitation, they are likely to transfer these positive emotions to their spouses (Bakker and Demerouti, 2009), which may strengthen spouses’ well-being (Bakker et al., 2009a). In addition, employees who benefit from work-family facilitation are likely to have positive interactions with their spouses, thereby increasing spousal family satisfaction (Van Steenbergen et al., 2014; Liu et al., 2016). We thus propose the following hypothesis:

*Hypothesis 5*: Employee work-family facilitation is positively related to spousal family satisfaction.

Benevolent leaders provide employees with instrumental career and emotional support. This consistent leadership support may facilitate employees gaining more resources to use in managing their family roles, which may in turn have a significant positive impact on their spouses’ family satisfaction. We thus propose the following full mediation hypothesis:

*Hypothesis 6*: Employee work-family facilitation mediates the positive relationship between benevolent leadership and spousal family satisfaction.

## The Moderating Role of Spouse’s Need for Control

Employees that suffer from work–family conflict may be unable to complete their family obligations timely, and deliver at their own pace rather than according to previously made plans, leaving the spouse to continuously having to adapt to the employee’s work demands. Spouses with a high need for control of their environments may be even more impacted by this disarray since last minute changes to family schedules provide little opportunity to control the family environment (Rijk et al., 1998). Spouses with a high need for control may be more vulnerable to the negative effects of employee work–family conflict and may experience lower levels of family satisfaction. On the other hand, spouses with a low need for control may be more understanding of a disorganized family environment. As a result, they may be more willing to adjust their expectations, which may in turn decrease the negative effects of employee work–family conflict on spousal family satisfaction. We thus propose the following hypothesis:

*Hypothesis 7*: Spouse’s need for control moderates the negative relationship between employee work-family conflict and spousal family satisfaction, such that the relationship is stronger when the spouse’s need for control is higher.

We further expect that spouse’s need for control will moderate the indirect effect of authoritarian leadership on spousal family satisfaction *via* employee work–family conflict (i.e., moderated mediation). In other words, we predict a stronger negative indirect effect of authoritarian leadership on spousal family satisfaction through employee work–family conflict when spouses report a higher need for control.

*Hypothesis 8*: The spouse’s need for control moderates the indirect effect of authoritarian leadership on spousal family satisfaction through employee work-family conflict, such that the indirect effect is stronger when spouse’s need for control is higher.

## Materials and Methods

### Participants and Procedure

We collected data from a real estate company and two IT companies in a metropolitan city in northern China. Each company assigned a human resources (HR) employee to act as a research coordinator. The sample involved all departments in all three organizations, including logistics, research, and development, procurement, manufacturing, marketing and sales, logistics, administrative management, and finance. To increase the response rate, both the HR coordinator and the researchers used instant messenger to remind participants to fill in and submit the questionnaires after the questionnaires were distributed.

To reduce common method bias and provide empirical support for causality, we collected data in three waves. Further, data were collected from both employees and their spouses. In the first wave (T1), employees reported their demographic information, and employee perceptions of authoritarian and benevolent leadership. We received 283 valid questionnaires in T1 with a response rate of 88.4%. After 2months, we invited these employees to complete a questionnaire reporting their work–family conflict and work-family facilitation. We received 255 valid questionnaires in the second wave (T2). In the third wave (T3), we sent a data collection package to employees who completed T1 and T2 surveys. The package included a cover letter explaining the research purpose and procedures, a spouse questionnaire, and a blank envelope for spouses to return the questionnaire to ensure confidentiality. Each employee then took the package home and spouses filled out the survey reporting their family satisfaction and need for control.

We matched employee questionnaires with spouse questionnaires based on the codes placed on the envelopes. We received 223 matched questionnaires with a response rate of 87.5%. We excluded four blank and 12 incomplete questionnaires from the final sample, leaving 207 matched complete questionnaires. Among employees, 134 were female (64.7%) and 73 were male (35.3%). The majority had a bachelor’s degree (62.3%), 15% had a graduate degree, 16.4% reported having an associate degree, and 6.3% had a high school diploma. Their average age was 36.4 (*SD*=1.36). Among spouses, 73 were female (35.3%) and 134 were male (64.7%). The majority had a bachelor’s degree (51.7%), 19.8% had a graduate degree, 20.8% reported having an associate degree, and 7.7% had a high school diploma. Their average age was 38 (*SD*=1.35). Majority of the employee-spouse dyads were dual career couples (87.4%).

### Measures

Authoritarian leadership and benevolent leadership scales were originally developed in Chinese (Cheng et al., 2000), however, all other measures were originally created in English. We translated these measures into Chinese using back-translation procedures (Brislin, 1986). We used a six-point Likert scale (1=*strongly disagree*, 6=*strongly agree*) to assess all variables because due to the influence of Confucianism, Chinese people tend to choose the midpoint on surveys. Thus, we used a six-point Likert scale in an effort to obtain more accurate reactions from our research participants (Cheng et al., 2004; Zhang et al., 2015; Chen et al., 2019).

*Authoritarian leadership* (Time 1) was measured with a 13-item scale developed by Cheng et al. (2000). Sample items include “We have to follow the supervisor’s rules to get things done. If not, he or she punishes us severely,” “My supervisor keeps information to himself or herself,” and “My supervisor emphasizes that our group must have the best performance of all the units in the organization” (*α*=0.92).

*Benevolent leadership* (Time 1) was assessed with an 11-item measure developed by Cheng et al. (2000). Sample items include “My supervisor responds to my request and meets my personal needs,” “My supervisor takes good care of my family members as well,” and “Beyond work relations, my supervisor expresses concern about my daily life” (*α*=0.93).

*Work–family conflict* (Time 2) was measured with a five-item scale developed by Netemeyer and Mcmurrian (1996). Sample items are “The demands of my work interfere with our home and family life,” “The amount of time my job takes up makes it difficult to fulfill family responsibilities,” and “My job produces strain that makes it difficult to fulfill family duties” (*α*=0.91). This measure has been used extensively in previous studies (e.g., Mesmer-Magnus and Viswesvaran, 2005) and has shown acceptable psychometric qualities in Chinese contexts (e.g., Lu et al., 2009).

*Work-family facilitation* (Time 2) was measured with a five-item scale from Grzywacz and Marks (2000). This measure has been extensively used in the work-family facilitation literature (e.g., Wayne et al., 2004). Sample items are “The things you do at work help you deal with personal and practical issues at home,” “The skills you use on your job are useful for things you have to do at home,” and “Having a good day on your job makes you a better companion when you get home” (*α*=0.81).

*Spouse’s need for control* (Time 3) was assessed from the spouses’ perspective, with a four-item measure from Rijk et al. (1998). This scale focuses not only on the use of control but also on the opportunities to exert control. Sample items are “I always do my own planning,” “I always control over what I do and the way that I do it,” and “I always give orders instead of receiving them” (*α*=0.81).

*Spousal family satisfaction* was assessed from the spouses’ perspective using three satisfaction items from the Michigan Organizational Assessment Questionnaire (Cammann et al., 1979). This three-item family satisfaction scale has been widely employed in previous research and has demonstrated acceptable reliability and validity estimates (e.g., Carlson et al., 2010). A sample item is “Generally speaking, I am very satisfied with my family” (*α*=0.84).

*Control variables* (Time 1). Based on previous research findings (e.g., Zhang et al., 2012; Zhang and Tu, 2018) on differential gender roles in the family, and the impact of life stage on expectations of family and life obligations, we controlled for gender and age of employees and their spouses. Further, education may boost employees’ chances in attaining access to resources that decrease job strain and enrich their family lives. Thus, we controlled for education levels of both employees and their spouses.

## Results

### Measurement Model

We employed confirmatory factor analyses (CFAs) to test the discriminant validity of authoritarian leadership, benevolent leadership, work–family conflict, work-family facilitation, spousal family satisfaction, and spouse’s need for control. The authoritarian leadership scale included 13 items and the benevolent leadership scale had 11 items. Scales with many indicators can alter the ratio between the sample size needed and the number of parameters estimated (Sass and Smith, 2006; Williams et al., 2009). Item parceling can be employed to address this issue, which involves combining items to create parcels which are then used as indicators. Parceling facilitates obtaining item distributions that are normally distributed and improve model fit by reducing the magnitude of specific variances (Bandalos, 2002; Little et al., 2013). Similar to previous research (e.g., Ou et al., 2014; Cooper et al., 2018; Lam et al., 2018), we reported CFA results using the parceling approach with the item-to-construct-balance method by pairing the high-loaded item with the low-loaded item. Specifically, we combined 13 items of authoritarian leadership into seven items, and combined 11 items of benevolent leadership into six items. In total, we obtained 30 indicators for six latent constructs. The CFA results indicate that the theorized six-factor latent model demonstrates better model fit (*χ*^2^=874.50; *df*=480; *RMSEA*=0.07; *CFI*=0.92; *TLI*=0.91; *SRMR*=0.06) than all five alternative models shown in Table 1.

**Table 1:** Model fit results of confirmatory factor analyses (CFAs) using structural equation modeling.

Models	*χ* ^2^	*df*	*χ*^2^/*df*	Δ*χ*^2^	*RMSEA*	*CFI*	*TLI*	*SRMR*
**Proposed six-factor model**
(Authoritarian leadership, benevolent leadership, WFC, WFF, spouse need for control, and spousal family satisfaction)	701.00	362	1.94		0.07	0.91	0.90	0.06
**Alternative five-factor model**
(WFC and WFF combined)	1006.55	367	2.74	305.55^***^	0.10	0.83	0.82	0.11
**Alternative four-factor model**
(WFC and WFF combined; authoritarian leadership, and benevolent leadership combined)	1686.38	371	4.55	985.38^***^	0.14	0.66	0.62	0.14
**Alternative three-factor model**
(Authoritarian leadership, benevolent leadership, WFC, and WFF combined)	2157.13	374	5.77	1456.13^***^	0.16	0.55	0.501	0.15
**Alternative two-factor model**
(Authoritarian leadership, benevolent leadership, WFC, WFF, and spousal family satisfaction combined)	2416.43	376	6.43	1715.43^***^	0.17	0.47	0.43	0.16
**Alternative one-factor model**
(All study variables combined)	2789.30	377	7.40	2088.30^***^	0.18	0.37	0.32	0.17

*N*=207. All alternative models were compared with the theorized six-factor model.

*** *p*<0.001. wfc, work-family conflict; wff, work-family facilitation.

### Descriptive Statistics

Table 2 presents the means, SDs, and correlations among all study variables. Authoritarian leadership was positively related to employee work–family conflict (*r*=0.49, *p*<0.001). Benevolent leadership was positively related to employee work-family facilitation (*r*=0.51, *p*<0.001) and spousal family satisfaction (*r*=0.17, *p*=0.02). Employee work–family conflict was negatively related to spousal family satisfaction (*r*=−0.30, *p*<0.001), whereas employee work-family facilitation was positively related to spousal family satisfaction (*r*=0.29, *p*<0.001).

**Table 2:** Descriptive statistics and correlations.

Variable	*Mean*	*SD*	1	2	3	4	5	6	7	8	9	10	11	12
1. Gender	0.34	0.48												
2. Age	36.4	1.36	0.03											
3. Education	2.82	0.72	−0.04	−0.20^**^										
4. Gender (S)	0.64	0.48	−0.96^***^	0.00	0.01									
5. Age (S)	0.38	1.35	−0.17^*^	0.81^***^	−0.17^*^	0.21^**^								
6. Education (S)	2.78	0.81	−0.08	−0.12	0.46^***^	0.09	−0.07							
7. Authoritarian leadership T1	3.44	0.87	0.08	0.05	−0.12	−0.04	−0.01	−0.11	(0.92)					
8. Benevolent leadership T1	3.79	0.88	0.06	0.07	−0.10	−0.06	0.14	−0.03	−0.36^***^	(0.93)				
9. Work family facilitation T2	4.13	0.86	−0.07	0.18^*^	0.08	0.04	0.21^**^	0.13	−0.21^**^	0.51^***^	(0.81)			
10. Work family conflict T2	3.44	0.95	0.09	−0.05	−0.02	−0.10	−0.14^*^	−0.01	0.49^***^	−0.20^**^	−0.23^**^	(0.91)		
11. Need for control (S) T3	4.22	0.73	−0.28^***^	0.07	0.07	0.26^***^	0.15^*^	0.13	−0.09	0.17^*^	0.20^**^	−0.20^**^	(0.81)	
12. Family satisfaction (S) T3	4.20	0.88	−0.10	0.14^*^	0.14^*^	0.10	0.22^**^	0.16^*^	−0.08	0.17^*^	0.29^***^	−0.30^***^	0.43^***^	(0.84)

*N*=207. Cronbach’s alphas are shown on the diagonal. S, spouse; T1, time 1; T2, time 2; and T3, time 3.

* *p*<0.05,

** *p*<0.01,

*** *p*<0.001.

### Structural Model

We examined our hypotheses by using PROCESS (Hayes, 2013) in SPSS. Hypothesis 1 posits that authoritarian leadership is positively related to employee work–family conflict. As shown in Model 1 in Table 3, authoritarian leadership was positively related to employee work–family conflict (*b*=0.53, *p*<0.001) providing support for H1. Hypothesis 2 indicated that employee work–family conflict is positively related to spouse’s family satisfaction. As shown in Model 3 in Table 3, employee work–family conflict was negatively related to spousal family satisfaction (*b*=−0.26, *p*<0.001) providing support for H2. Hypothesis 3 suggested that employee work–family conflict mediates the negative relationship between authoritarian leadership and spousal family satisfaction. In support of H3, authoritarian leadership indirectly affected spousal family satisfaction *via* employee work–family conflict (*indirect effect*=−0.14, 95% CI=[−0.23, −0.06]), since the simulated 95% CI (based on bootstrapping with 5,000 random samples) did not contain zero, providing support for the mediating effect. Thus, H3 was supported.^1^

**Table 3:** Direct, indirect, and interactive effects of authoritarian leadership, benevolent leadership, work–family conflict, work-family facilitation, and spouses’ need for control on spousal family satisfaction.

Variables	Employee WFC	Employee WFF	Spousal family satisfaction		
	Model 1	Model 2	Model 3	Model 4	Model 5
Intercept	2.08^*^ (0.61)	1.70^*^ (0.58)	3.98^***^ (0.60)	2.15^**^ (0.67)	3.06^***^ (0.55)
Gender (employee)	−0.28 (0.44)	−0.78 (0.44)	−0.53 (0.43)	−0.17 (0.49)	−0.16 (0.40)
Age (employee)	0.08 (0.08)	0.05 (0.07)	−0.00 (0.08)	0.01 (0.08)	−0.02 (0.07)
Education (employee)	−0.02 (0.10)	0.16 (0.09)	0.17 (0.10)	0.14 (0.10)	0.16 (0.09)
Gender (spouse)	−0.35 (0.44)	−0.64 (0.44)	−0.46 (0.42)	−0.04 (0.49)	−0.28 (0.40)
Age (spouse)	−0.17 (0.08)	0.07 (0.07)	0.16 (0.08)	0.15 (0.08)	0.13 (0.08)
Education (spouse)	0.07 (0.09)	0.14 (0.08)	0.10 (0.09)	0.07 (0.09)	0.08 (0.08)
Authoritarian leadership	0.53^***^ (0.07)		0.10 (0.08)		0.07 (0.08)
Benevolent leadership		0.50^***^ (0.06)		0.09 (0.08)	
Employee work–family conflict			−0.26^***^ (0.07)		−0.17^**^ (0.07)
Employee work-family facilitation				0.17^*^ (0.08)	
Spouses’ need for control					0.30^***^ (0.06)
Employee work–family conflict×Spouses’ need for control					−0.13^*^ (0.05)
*R* ^2^	0.26	0.34	0.18	0.17	0.30

*N*=207. SE is reported in the parentheses.

* *p*<0.05.

** *p*<0.01.

*** *p*<0.001.

Hypothesis 4 suggested that benevolent leadership is positively related to employee work-family facilitation. As shown in Model 2 in Table 3, benevolent leadership was positively related to employee work-family facilitation (*b*=0.50, *p*<0.001) providing support for H4. Hypothesis 5 posited that employee work-family facilitation was positively related to spousal family satisfaction. As shown in Model 4 in Table 3, employee work-family facilitation was positively related to spousal family satisfaction (*b*=0.17, *p*=0.05) providing support for H5. Hypothesis 6 states that employee work-family facilitation mediates the positive relationship between benevolent leadership and spousal family satisfaction. In support of H6, benevolent leadership indirectly affected spousal family satisfaction *via* employee work-family facilitation (*indirect effect*=0.08, 95% CI=[0.002, 0.18]), since the simulated 95% CI did not contain zero, providing support for the mediating effect. Thus, H6 was supported.^2^

Previous research suggests that work–family conflict and work-family facilitation are two relatively independent processes (Chen and Powell, 2012). Thus, we also explored whether work–family conflict mediates the relationship between benevolent leadership and spouse’s family satisfaction and whether work-family facilitation mediates the relationship between authoritarian leadership and spouse’s family satisfaction. The results demonstrated that work–family conflict did not mediate the relationship between benevolent leadership and spouse’s family satisfaction (indirect effect=0.04, 95% CI=[−0.0003, 0.10]), as the simulated 95% CI contained zero. Work-family facilitation also did not mediate the relationship between authoritarian leadership and spouse’s family satisfaction (indirect effect=−0.04, 95% CI=[−0.10, 0.003]), as the simulated 95% CI contained zero.

**Figure 2:**
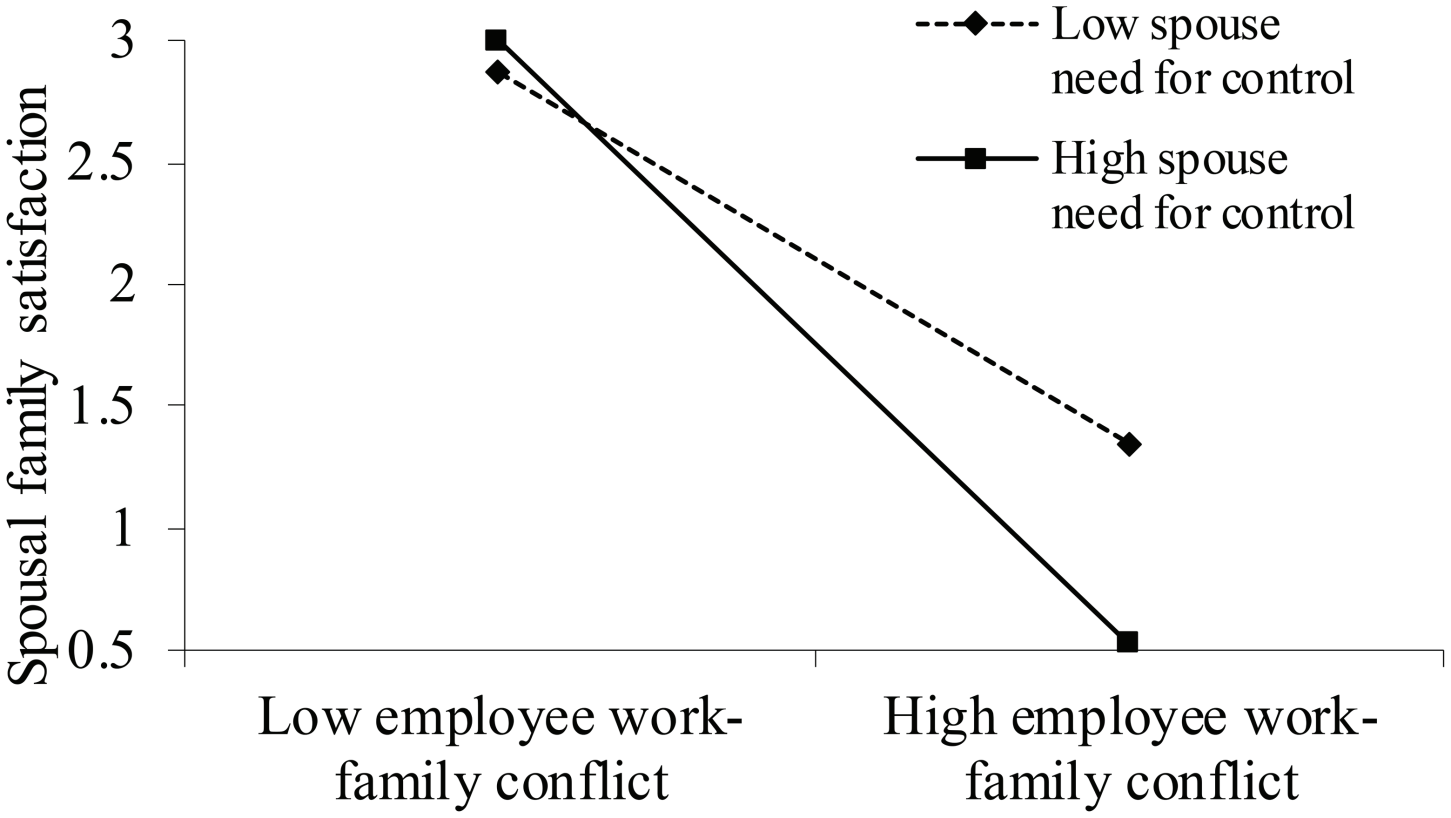
The moderating effect of spouses’ need for control on the relationship between work–family conflict and spousal family satisfaction.

Hypothesis 7 expected that spouse’s need for control would moderate the negative relationship between employee work–family conflict and spousal family satisfaction. Hypothesis 8 posited that spouse’s need for control would moderate the indirect effect of authoritarian leadership on spousal family satisfaction through employee work–family conflict. We first mean-centered employee work–family conflict and spouse’s need for control scores and then used PROCESS in SPSS to examine the moderation effect as well as the moderated mediation. As shown in Model 5 in Table 3, the interaction between employee work–family conflict and spouse’s need for control was negative and significant (*b*=−0.13, *p*=0.02). The results of the simple slope test suggest when spouse’s need for control was high, the relationship between employee work–family conflict and spousal family satisfaction was significantly negative (*b*=−0.30, *p<* 0.001). When spouse’s need for control was low, the relationship between employee work–family conflict and spousal family satisfaction was negative but not significant (*b*=−0.04, *p*=0.64; Figure 2), providing support for H7. The index of moderated mediation (IMM) was significant (IMM=−0.07, 95% CI = [−0.13, −0.01]) as the simulated 95% CI did not contain zero (Hayes, 2015). Authoritarian leadership had a stronger indirect effect on spousal family satisfaction *via* employee work–family conflict when spouse’s need for control was high (*indirect effect*=−0.17, 95% CI=[−0.28, −0.08]) compared to when spouse’s need for control was moderate (*indirect effect*=−0.10, 95% CI=[−0.18, −0.03]) or low (*indirect effect*=−0.02, 95% CI=[−0.11, 0.06]). Thus, H8 was supported.

## Discussion

This study offers one of the first attempts to map out both the positive and the negative leadership impacts on employee spouses. Rooted in COR theory and crossover theory, we investigate whether and how authoritarian leadership and benevolent leadership affect the family satisfaction of employees’ spouses. Using a three-wave, multiple-source (employee-spouse dyads) research design, we found that authoritarian leadership triggers employee work–family conflict, which in turn decreases spousal family satisfaction. In contrast, benevolent leadership promotes employee work-family facilitation, which helps improve spousal family satisfaction. Further, our results identify spouse’s need for control may strengthen the negative effect of employee work–family conflict on spousal family satisfaction. Specifically, when employees experience work–family conflict, family satisfaction decreases faster for spouses who report higher levels of need for control compared with those who report a lower need for control.

### Theoretical Implications

Our research contributes to leadership and work–family literatures by providing a more nuanced perspective of leadership’s impact on family, extending the focus from the employee to spousal outcomes. We respond to previous calls for integrating leadership theory with work–family literature (Major and Litano, 2016) to provide a more complete picture of the relationship between leadership and employees’ family life. Findings provide support for the nomological link between authoritarian leadership, benevolent leadership, and spousal family satisfaction. We integrate COR theory and crossover theory to demonstrate that authoritarian and benevolent leadership have far-reaching differential impact beyond the work domain, which may affect the well-being of employees’ spouses. We offer a new perspective for better understanding the link between leadership, employees, and employees’ spouse. It is vital for organizations to pay attention to the spouses’ well-being because employees’ family relations at home may directly impact employee attitudes and effectiveness at work. Further, the majority of the married dyads in our sample were dual career couples, and the negative impact of authoritarian leadership in one organization may also negatively influence effectiveness in the spouse’s workplace through spousal family dissatisfaction. Previous research has found that negative family emotions are hard to leave at home when employees enter the workplace whereas spousal support at home may enhance employee performance (Madjar et al., 2002). ten Brummelhuis et al. (2013) suggest that studying the home domain may inform employee’s unscheduled absence from work as well as employee absence frequency and duration. Therefore, the positive impact of benevolent leadership in one organization may have a positive spillover effect on the spouse’s employer as well.

Further, this study enriches our understanding of the intermediate mechanisms through which leadership impacts employees’ family domain. Findings demonstrate that both work–family conflict and work-family facilitation act as intermediate variables in bridging the work and family domains. A growing number of studies have acknowledged the impact of leadership behaviors on employees’ family domain (e.g., Zhang et al., 2012; Tang et al., 2016; Zhang and Tu, 2018), yet only a few have simultaneously studied work–family conflict and work-family facilitation as mediators (Li et al., 2017). Our study provides further support that work–family conflict and work-family facilitation represent significant linking mechanisms in the relationship between leadership and employees’ family well-being.

One important aspect of our time lapsed results that bears further mention is that our study advances our knowledge of how spousal characteristics can impact employees’ family well-being. We modeled spouse’s need for control as a boundary condition and results provide support for a significant moderating effect. Results suggest that the negative indirect effect from authoritarian leadership to spousal family satisfaction is amplified for spouses with higher levels of need for control. This is an important finding which may have far-reaching consequences beyond employee’s home to the employee’s as well as the spouse’s work settings.

Finally, our research advances existing research on the effects of work–family conflict on Chinese family life, with important considerations for work-family research in the Western context. Previous research has consistently demonstrated that collectivistic cultures encourage employees to work hard to increase overall family wealth (Lu et al., 2015). Therefore, spouses of Chinese employees may be more likely to expect and accept employees putting considerable time and effort into work as compared with spouses in individualistic cultures, where spouses may perceive routinely working overtime while shirking family responsibilities as being unacceptable (Zhang et al., 2013). This is an important emerging research area given dual-earner couples are on the rise (Petriglieri, 2019). According to Pew Research Center (2015), 63% of the couples with children in the United States are dual-career couples and this number is even higher in the EU. This research further suggests that for working parents, attitudes toward balancing their job and their family life are highly correlated with their experiences as parents. Given the widespread and increasing prevalence of dual-career couples, a more in-depth understanding of work-family nuances, specifically the moderating effects for established relationships should aid both employees and spouses, as well as organizations looking to implement training programs and supportive practices in support of dual careers.

### Practical Implications

Practical implications of the present study are attainable because leadership behaviors are within the control of organizations. This study demonstrates the positive impact of benevolent leadership on both the employee (through work-family facilitation) and the spouse. Although our study was based in China, previous research supports the positive impact of benevolent leadership on LMX and affective organizational commitment in the Western business context (Pellegrini et al., 2010). Findings also demonstrate the negative impact of authoritarian leadership on both the employee (through work–family conflict) as well as the employee’s spouse. Given the negative dual impact of authoritarian leadership, what can organizations do?

First, organizations should carefully monitor and manage leaders’ authoritarian tendencies to avoid or reduce their negative dual impact on employees and spouses, who may also be employees of another business organization. Leaders need to be trained to understand the potential far-reaching negative impacts of authoritarian behaviors. Authoritarian leaders should be encouraged to reflect on and recognize their behaviors, learn to decrease their tight control over employees, and adopt benevolent leadership as a model by providing more personalized career and personal support to employees. Authoritarian leaders could attend leadership training programs geared toward self-awareness and learning to become more well-rounded leaders who can help employees thrive both on and off-the-job domains.

Second, when employees experience authoritarian leadership, they should be particularly mindful of not carrying their work-related stressors into the family domain. Employees might also find counseling helpful to obtain guidance in coping strategies that they can use to maintain harmony between their work and family lives (Bodenmann et al., 2008). These counseling sessions could be subsidized by employers to encourage employees build the necessary skills for an effective work-life balance. Employees and their partners should recognize that their relationship may be affected by positive and negative spillover from each other’s work domains.

Third, organizations need to expend more time and effort and take timely measures to reduce work–family conflict spillover to the family. They should also do more to support dual careers since the negative spillover from work–family conflict may be amplified for employees whose partners also work outside the home. The continuing growth of dual careers has implications for attraction, retention, and overall talent management. Companies that are at the forefront of recognizing the broader impact on diversity in their workforce and improved sense of work-life balance for employees and their spouses, may reap first-mover benefits in creating a work environment that attracts and retains the best talent. For example, organizations may create a resource-abundant work environment by providing flexible work schedules, infusing jobs with greater autonomy, and promoting practices that encourage employees to be empowered (Ford et al., 2007). In these settings, employees would be more likely to attain work-family facilitation, which would in turn support employees’ and their spouses’ well-being (Taylor et al., 2009; Carlson et al., 2011). In turn, supportive practices should increase the organization’s competitiveness in talent attraction and retention because millennials view work-life balance more as integration, rather than equilibrium, and they actively seek work roles that allow them to thrive outside of work (Miller and Yar, 2019; Alesso-Bendisch, 2020).

### Limitations and Future Directions

Despite the aforementioned strengths of the present study, like most research studies, one should interpret the results within the context of the study’s limitations. First, results showed that authoritarian leadership is positively related to employee work–family conflict, while work–family conflict is negatively related to spousal family satisfaction. However, work–family conflict may be acting as a suppressor variable masking an association between authoritarian leadership and spousal family satisfaction (MacKinnon et al., 2000). We followed the suggestions from MacKinnon et al. (2000), Eisenberger et al. (2010), and Schneider et al. (2005) and did not require a significant direct relationship between the independent variable and the dependent variable as a requirement to establish full mediation. Future studies should explore additional mediators to build a more integrated network of relationships between authoritarian leadership and employee family life. Future researchers could also investigate emotions (e.g., anger or gratitude) and social interactions (e.g., supportive or undermining employee behaviors at home) to gain a deeper understanding of how employee emotions and behaviors triggered by authoritarian or benevolent leadership may transfer to spouses, so that organizations could design specific intervention strategies.

Further, we recommend that future studies explore the effects of leadership on job attitudes and job performance of employees’ spouses, as the number of dual-earner couples is growing rapidly (Greenhaus and Powell, 2012). In dual-earner couples, the potential for stress and benefits for one partner may crossover to the spouse’s work domain. For example, Carlson et al. (2017) found that employee use of mobile devices for work during family time increase spouse’s family-to-work conflict, which subsequently show a negative spillover for the spouse in reduced spousal job satisfaction and performance. Further investigation of this dynamic crossover relationship could help dual-career partners as well as business organizations in strategizing supportive practices to foster a more inclusive and prosperous work environment.

Third, we investigated the role of the spouse’s need for control in the negative relationship between employee work–family conflict and spousal family satisfaction. Future research might explore other key resource variables, such as resilience or openness to change. An in-depth investigation of individual differences would increase our understanding of how these variables function in alleviating or exacerbating work-family spillovers. For example, conscientiousness may help individuals apply resources efficiently to achieve goals, while emotional stability may protect them from resource loss (Perry et al., 2010).

Finally, our data were collected from employees and their spouses in the Chinese work context. Future research should examine the generalizability of these findings in similar work contexts where benevolent and authoritarian leadership are prevalent, such as Asia Pacific, the Middle East, and Latin America.

## Conclusion

We demonstrated that authoritarian leadership and benevolent leadership have a long reach with significant impact on employee family domain. We find that authoritarian leadership increases employee work–family conflict, which then decreases spousal family satisfaction. Moreover, spouse’s need for control further strengthens this negative impact. Based on these findings, the costs of business organizations not taking timely action in eliminating authoritarian leadership behaviors, could have far-reaching societal impact on family structures. Thus, we suggest that we interpret this study’s findings remembering the old adage “An ounce of prevention is worth a pound of cure,” and business organizations at the very least should start encouraging benevolent leadership as they work towards eliminating authoritarian practices.

## Data Availability Statement

The original contributions presented in the study are included in the article/supplementary material, further inquiries can be directed to the corresponding author.

## Author Contributions

LY designed and drafted this study. MX and LY conducted the statistical analysis, interpreted data, and drafted the initial manuscript. EP is responsible for the final paper review and editing. All authors critically reviewed and approved the final version of this manuscript.

## Conflict of Interest

The authors declare that the research was conducted in the absence of any commercial or financial relationships that could be construed as a potential conflict of interest.

## Publisher’s Note

All claims expressed in this article are solely those of the authors and do not necessarily represent those of their affiliated organizations, or those of the publisher, the editors and the reviewers. Any product that may be evaluated in this article, or claim that may be made by its manufacturer, is not guaranteed or endorsed by the publisher.

## Footnotes

^1^We found that the relationship between authoritarian leadership is positively related to employee work–family conflict, while employee work–family conflict is negatively related to spousal family satisfaction. Work–family conflict may act as a suppressor variable in the relationship between authoritarian leadership and spousal family satisfaction. Results suggest that authoritarian leadership is not significantly related to spousal family satisfaction (*b* = 0.08, *p* > 0.05). MacKinnon et al. (2000) indicate that the relationship between X and Y may not be significant due to suppressor effects. Eisenberger et al. (2010) suggest that the relationship between X and Y may be hard to detect due to distal effects not having sufficient power, while the mediation effect may be detected when proximal effects have enough power. Further, numerous researchers suggested that it is not necessary to show a direct relationship between X and Y in mediation models (Collins et al., 1998; Shrout and Bolger, 2002; Schneider et al., 2005). We follow previous recommendations in supporting Hypothesis 3.

^2^We found that benevolent leadership is not significantly related to spousal family satisfaction (*b* = 0.09, *p* > 0.05). Based on previous research recommendations on mediating effects (Collins et al., 1998; Shrout and Bolger, 2002; Schneider et al., 2005; Eisenberger et al., 2010) H6 was supported.
